# Computational Analysis of Protein Structure Changes as a Result of Nondeletion Insertion Mutations in Human *β*-Globin Gene Suggests Possible Cause of *β*-Thalassemia

**DOI:** 10.1155/2019/9210841

**Published:** 2019-05-29

**Authors:** Talal Qadah, Mohammad Sarwar Jamal

**Affiliations:** ^1^Department of Medical Laboratory Technology, Faculty of Applied Medical Science, King Abdulaziz University, Jeddah, Saudi Arabia; ^2^Hematology Research Group, King Fahd Medical Research Center, King Abdulaziz University, Jeddah, Saudi Arabia

## Abstract

Beta-thalassemia is described as a group of hereditary blood disorders characterized by abnormalities in the synthesis of beta chains of hemoglobin. These anomalies result in different phenotypes ranging from moderate to severe clinical symptoms to no symptoms at all. Most of the defects in hemoglobin arise directly from the mutations in the structural *β*-globin gene* (HBB). *Recent advances in computational tools have allowed the study of the relationship between the genotype and phenotype in many diseases including *β*-thalassemia. Due to high prevalence of *β*-thalassemia, these analyses have helped to understand the molecular basis of the disease in a better way. In this direction, a relational database, named HbVar, was developed in 2001 by a collective academic effort to provide quality and up-to-date information on the genomic variations leading to hemoglobinopathies and thalassemia. The database recorded details about each variant including the altered sequence, hematological defects, its pathology, and its occurrence along with references. In the present study, an attempt was made to investigate nondeletion mutations in the* HBB* picked up from HbVar and their effects using the in silico approach. Our study investigated 12 nucleotides insertion mutations in six different altered sequences. These 12 extra nucleotides led to the formation of a loop in the protein structure and did not alter its function. It appears that these mutations act as ‘silent' mutations. However, further* in vitro* studies are required to reach definitive conclusions.

## 1. Introduction

Hemoglobinopathies are genetic disorders caused by single-gene variations in the *α*-like and *β*-like human globin gene clusters. These are the most common inherited disorders in humans with nearly 7% of the human population acting as carriers of the mutations in globin genes. Single nucleotide substitutions in the coding or regulatory regions of these genes can lead to varying degrees of defects in their expression [1]. The* HBB* gene belongs to the *β*-globin gene cluster that encodes *β*-globin polypeptide. It is located in the short arm of chromosome 11 and contains two introns and three exons. Molecular defects in human* HBB* may result in structural defects causing abnormality in hemoglobins, such as HbS, HbC, and HbD, or may result in absence or reduced synthesis of *β*-globin chains causing *β*-thalassemia [2]. Mutations in the* HBB* may involve substitution, deletion, or insertion of one or multiple nucleotides within the gene or its flanking regions resulting in anemia and low RBC production [3]. *β*- Thalassemia is inherited as an autosomal recessive trait and its clinical manifestation can be divided as thalassemia major, intermedia, and thalassemia minor (trait) [4]. Some mutations in the* HBB* lead to complete inactivation of the gene resulting in the absence of *β*-globin chains (*β*^0^), in turn, leading to the most severe form of thalassemia. Other mutations allow production of *β*-globin chains in varying proportions leading to *β*^+^ thalassemia. This case is most commonly found in the Middle East, Central Asia, Mediterranean countries, India, and southern China and in some parts of Africa and South America [5]. It is one of the most common genetic disorders caused by point mutations in Saudi Arabia causing variable phenotypic effects. These phenotypic severities may arise from defects in transcription, RNA processing, or translation of the HBB gene [6]. The most common mutations in most Arab-populated countries include IVSI-110(G>A), IVSI-1(G>A), IVSI-6(T>C), IVSII-1(G>A), IVSI-5(G>C), codon 5(-CT), and codon 39 (C>T) [7].

Due to the high prevalence of variable phenotypes of thalassemia and striking heterogeneity of its molecular defects, various strategies were employed to investigate the molecular mechanisms of this disease. Due to recent advances in computational tools, in silico analysis has become one of the chosen methods to investigate links between genomic and resulting phenotypic characteristics in thalassemia. HbVar (http://globin.cse.psu.edu/globin/hbvar/) is the oldest and most appreciated database of hemoglobin variants and thalassemia mutations established in 2001 [8]. It is a locus specific database, which was developed as a combined academic effort to keep a record of hemoglobin variants, new data entries, updates, and corrections. It provides high quality and up-to-date information on the genomic variations, associated phenotypic and hematological effects, pathology, frequency of different mutations, ethnic prevalence, and references [9]. HbVar has become a primary resource for the research community working on globin proteins and for physicians dealing with patients with hemoglobinopathies, to help them with making proper diagnoses.

The objective of this study is to investigate the effects of insertion mutations in the* HBB* exons using the in silico approach. We aimed to search the HbVar database to select sequences with uncharacterized insertion mutations and study their effects on the structure and function of *β*-globin protein.

## 2. Methodology

To perform this study, data from the HbVar database (http://globin.bx.psu.edu/hbvar) were used. Using this database, we identified uncharacterized* HBB* sequences containing nondeletion mutations and picked them up for in silico investigation. The sequence of a wild* HBB* (gene ID 3043) was taken as reference (https://www.ncbi.nlm.nih.gov/gene/?term=3043). The potential 5' and 3' sites in the gene sequences were identified using the Human Splicing Finder software [10]. These predictions were compared and multiple sequence alignment was performed using the available web server. An online translation tool (https://web.expasy.org/translate/) was used to translate the nucleotide sequences into amino acids, and the types of mutations as well as their respective positions were noted. Mutations located in the defined donor and acceptor splice sites were included here. The relative strength of the sites obtained from the bioinformatics tool was given values between 0 to 100. Splice sites with high value were considered functional. Homology modeling of the wild type and mutant sequences was performed to compare the 3D structures of the proteins using the SWISS-MODELER [11].

## 3. Molecular Dynamic Simulation

The molecular dynamic simulation of the wild type and mutant proteins was performed using the GROMACS software [12]. The force field used for simulation was GROMOS96 53A6. The model structures (wild type and mutants) were solvated with water molecules in an octahedral box. Sodium ions (Na^+^) were added for neutralization. The solvated systems were then subjected to 5000 steps of energy minimization using the steepest descent method to remove the steric clashes. Convergence was achieved in the energy minimization when the maximum force was smaller than 1000 kJ mol^−1^ nm^−1^. The NPT ensemble was performed for 1000 ps at 300 K. The production simulation was executed at 300 K for 30 ns for the wild type and mutant proteins. Protein visualization and superimposition were performed using PyMOL software (https://pymol.org/2/). Root mean square deviation was analyzed using the PyMOL align module.

## 4. Results

**Figure 1:**
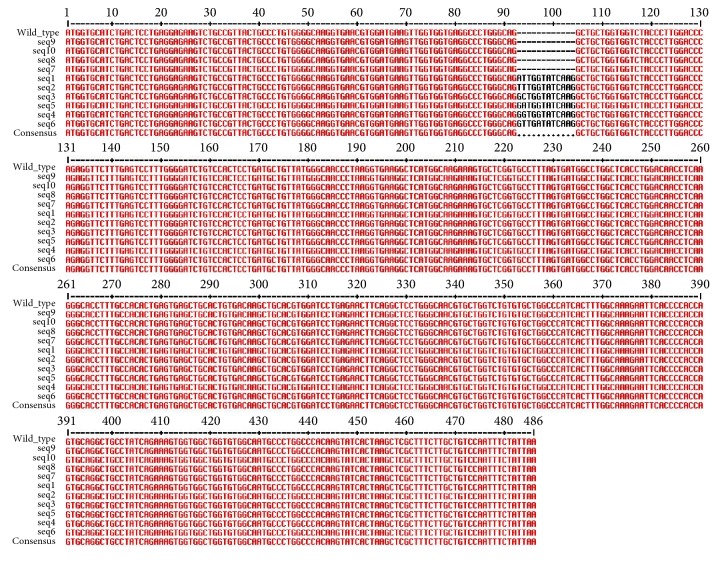
Multiple sequence alignment of the reference sequence and the multiple sequences, ATC, ATT, ACT, picked up from the HbVar database (after removing the introns). Twelve nucleotides have been inserted from position 93 to 104 in sequence numbers 1, 2, 3, 4, 5, and 6.

Multiple sequence alignment between reference and mutated gene sequences showed that at least 12 nucleotides have been inserted in the sequences 1, 2, 3, 4, 5, and 6 from position 93 to 104 as compared to the wild type (Figure 1). The inserted nucleotides showed maximum variation in the positions 93 and 94. In sequence 1 and sequence 2, adenine and thymine were inserted at position 93, respectively, whereas guanine appeared at position 93 in sequences 3, 4, 5, and 6. At position 94, thymine was inserted in sequences 1, 2, and 6, cytosine in sequence 3, guanine in sequence 4, and adenine in sequence 5. From position 95 to 104, the six sequences presented no variation among the inserted nucleotides, except for sequence 6 which had adenine inserted at position 97, instead of guanine like the remaining sequences.

**Figure 2:**
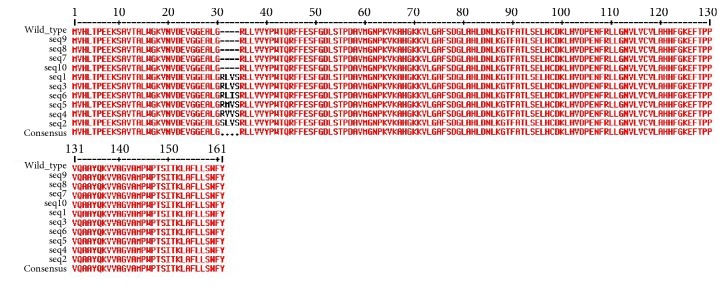
Protein sequence alignment of wild type and multiple sequences. Insertion of four amino acids has taken place at positions 31 to 34.

Variations among these inserted nucleotides gave rise to changes in amino acids in protein sequences. Twelve nucleotides in total gave rise to four amino acids where different variants have been identified (Figure 2 and Table 1).

**Figure 3:**
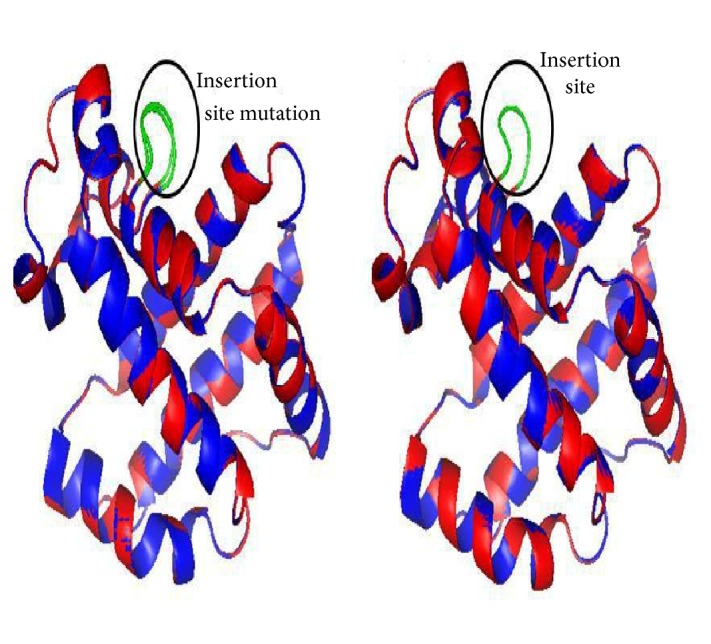
3D structures of wild type (red color) and mutant type (blue color) of the HBB protein. Insertion sequence has been differentiated in green in the provided figures.

The construction of the 3D structure of the HBB protein from the given mutated sequences showed that the inserted amino acids formed a loop structure (secondary structure) in the protein. The homology modeling of the wild type and mutant type HBB protein showed that the mutated segment did not form any well-defined secondary structure. The mutated segment formed a loop and connected two *α*-helical chains (Figure 3).

**Figure 4:**
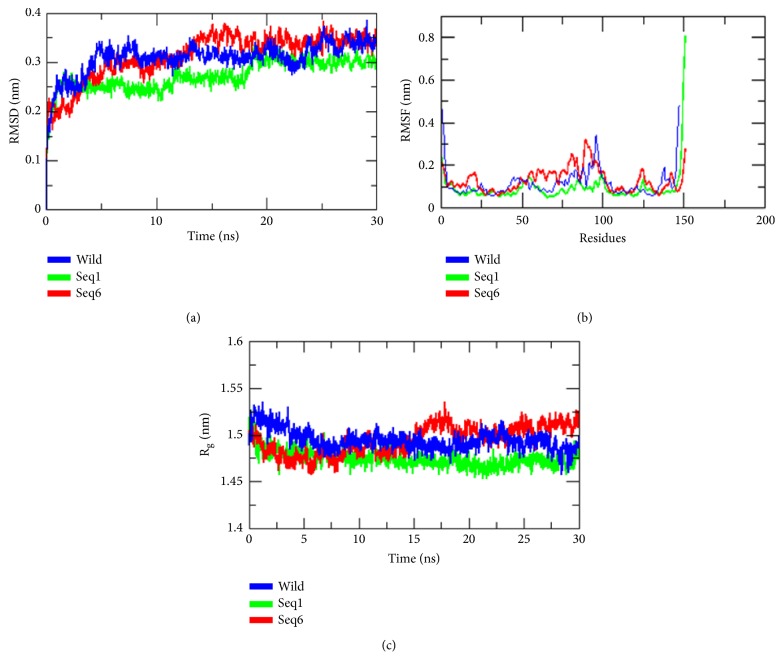
Molecular dynamic simulation: (a) root mean square deviation; (b) root mean square fluctuations; (c) radius of gyration of C*α* atoms of wild type and mutant proteins; time at 300 K.

**Figure 5:**
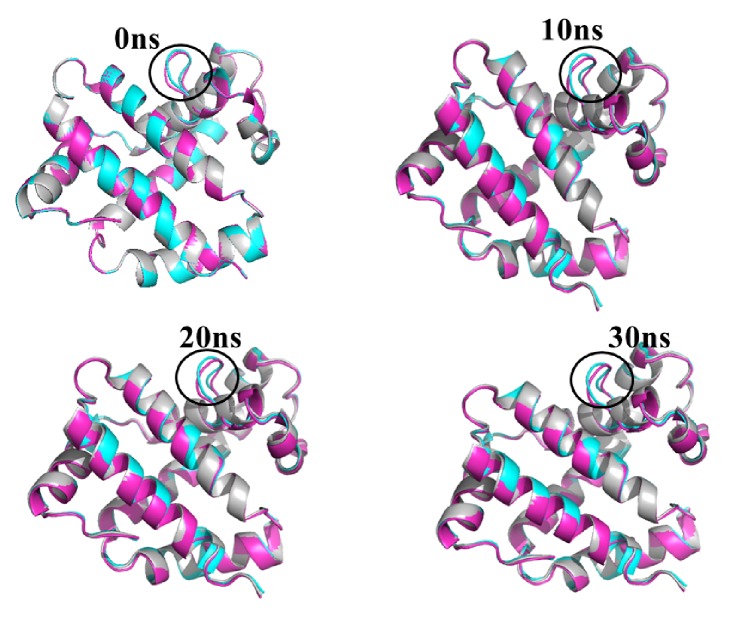
Conformation of model structures at 0ns, 10ns, 20ns, and 30ns. Cyan (Seq1), Magenta (Seq2), and Black brown (wild).

The stability and properties of the wild type and its mutant structures were studied by explicit solvent MD stimulation. Root mean square deviations have been calculated between different structures to form a complete picture of deviation in the structures of mutated proteins from the wild type (Table 2). The root mean square deviation (RMSD) analysis not only reflects the change of protein backbone versus simulation time, but also indicates the divergence of two structures. The RMSD of the homolog became stable at 30ns. The RMSD value of the wild type was 0.27 nm (Figure 4(a)). This result indicated that an accepted structure was obtained by the simulation that was reliable for further analyses. The root mean square fluctuation (RMSF) reflects the mobility of a certain residue around its mean position, which is another tool for studying the dynamics stability of the system. Although there were some deviations among the trajectories (especially in the loop region), the present data suggested less fluctuations, which further highlighted the reliability of the modeled structure (Figure 4(b)). The RMSF analysis can be used as a tool to describe local flexibility differences among residues throughout the MD simulation 62. According to Figure 4(b), the wild type protein and Seq6 showed an overall higher degree of flexibility when compared to the mutants. A difference in RMSF value was seen on residues 76-91. The wild type and Seq6 proteins showed a fluctuation of 0.32nm, while the fluctuation at the same position on Seq1 was 0.1nm, thus indicating a flexibility loss. Whereas residues 54–80 showed fluctuation values ranging from 0.15nm to 2.0nm in the wild type, while in the Seq1 and Seq6, these fluctuation values ranged from 0.09nm to 0.10nm. These results suggested that Seq1 affected the flexibility of the protein. This flexibility loss may affect protein function. Surprisingly, these fluctuations were found in the region away from the insertion or mutation site. However, Seq6 was found to have some fluctuation at this site as compared to the wild type and Seq1 (Figure 4(b)). We also analyzed the radius of gyration (Rg) values in the simulation. Rg is an indicator of structure compactness and overall dimension of the protein. It explains how regular secondary structures are compactly packed into the 3D structure of a protein. If a protein is folded well, it will likely maintain a relatively steady value of Rg, whereas it will change over time for unfolded proteins [13]. We found a stable Rg for Seq1 as compared to the wild type and Seq6 (Figure 4(c)). Low value of Rg for Seq1, as compared to the wild type and Seq6, suggested tight packing of these structures, making them relatively stable. The conformation of the modeled structure of Seq1 with different times, from 10ns, 20ns, and 30ns, was found to be similar. However, a slight difference has been observed at the insertion site (Figure 5).

## 5. Discussion

Constructing a relationship between the genotype and phenotype experimentally is an important aspect of research [14], but it can prove to be highly difficult, in particular, when studying a large number of subjects. The in silico analysis provides a solution here. It helps researchers analyze enormous amounts of data in biology to narrow down the positive leads that can be further analyzed experimentally for validation. This saves an extensive amount of labor, time, and costs. In silico analysis of large number of mutations is also easier and faster to accomplish, as this type of investigation is performed by comparing and studying alterations in the nucleotide and/or amino acid sequences with the wild/native type and then correlating these alterations with the changed phenotypes [15, 16, 16].

Generally, *β*-thalassemia affects the people of the Gulf, Middle Eastern, and Mediterranean regions [17]. Especially in Saudi Arabia, *β*- thalassemia is prevalent, though there are variations in the frequency of the gene and in the type of mutations [18]. Many previous studies have screened and reported different mutations in the* HBB* and their frequencies in the Saudi population [19–21].

Most of the mutations affecting the expression of the* HBB* are linked to the gene physically and form different alleles of the gene, but some mutations that affect the gene expression, while also segregating the* HBB *cluster, have also been identified [22, 23]. Although the* HBB* is well characterized, some mutations in this gene recorded in the HbVar database are poorly understood and have not been properly studied before. In this study, we picked up such mutations and performed in silico analysis to understand their effect on the protein structure and function.

The nondeletion forms of defects in the* HBB* account for a large number of the *β*-thalassemia alleles [24]. These include small insertions and single nucleotide substitutions or deletions within the gene or its nearby sequences [23]. Some of the alleles of *β*-thalassemia are very mild, in that carriers (heterozygotes) of such alleles are almost normal with no apparent signs of the disease, except for imbalanced synthesis of globin chains [25, 26]. One of the fairly common ‘silent' mutations in the Mediterranean population is 101 C→T. It interacts with other more severe mutations of *β*-thalassemia to cause milder forms [24]. Other ‘silent' mutations have been reported in the 5' UTR region of the* HBB* [25, 26].

The present study aimed to understand the nondeletion mutations using in silico tools. We identified 6 different sequences carrying insertion mutation of 12 nucleotides from position 93 to 104 in the gene sequence. Variations also occurred among the inserted nucleotides among these 6 different sequences. In silico analysis showed that these inserted nucleotides translated into 4 additional amino acids. These additional amino acids acquired a loop formation in the 3D structure of the protein (Figure 3). The difference in amino acids did not show any variation in the secondary loop structure acquired by these amino acids, but the molecular dynamics simulations presented evidence of effects caused by these mutations on the overall protein flexibility. The RMSF analysis showed a high degree of flexibility in the wild type protein and Seq6 in comparison to other mutant forms (Figure 4(b)). Additionally, significant flexibility loss was seen in the Seq1 mutant form, especially in residues from positions 54-80 and 76-91. Though this effect was seen far from the site of mutation in Seq1, it is quite possible that insertion of new amino acids may likely disturb the internal environment of the protein, resulting in a whole new set of interactions between amino acids, which in turn, might have affected protein flexibility. This loss in flexibility may result in the loss of function of the protein. Studies suggest that a change in protein structure and, consequently, in function could be because of genetic variation in distal effect, because of the temporal effect due to folding of the protein sequence culminating into the final protein structure. This particularly happens because of the change in the properties related to the physicochemical, such as hydrophobicity, charge, and geometry due to the side chain of the amino acid residues. If such changes occur at critical sites, such as catalytic positions or important interacting sites called interfaces, then it is likely the reason for disease causing variations, which further tend to destabilize various hydrogen bonds and the salt bridge [25–28]. To further prove the effects of these insertion mutations on protein structure and function, more in depth analysis is required.

Furthermore, the studies on radius of gyration of C*α* atoms of the wild type and mutant proteins depicted in the 3D structure of the Seq1 mutant protein, are seen to be more compact and stable, as compared to the wild type and Seq6 proteins. This data suggests that the insertion mutations in HBB protein might be affecting its overall structure and function as shown in Seq1 and Seq6, but more intensive studies are required to fully understand the scope of these effects. We are yet to determine how, if at all, these mutations affect the flexibility of HBB protein and whether this loss affects protein function and to what extent. In vitro studies will further assess the functional behavior of mutated proteins.

## Figures and Tables

**Table 1:** Variation in the protein sequences.

Amino Acids	Sequence_ID
R31	Seq1, Seq3, Seq 4, Seq5, Seq6
S31	Seq2
L32	Seq1, Seq2, Seq3, Seq6
M32	Seq5
V32	Seq4
V33	Seq1, Seq2, Seq3, Seq 4, Seq5
I33	Seq6

**Table 2:** Root mean square deviation (RMSD) between the model structures of the sequences.

Model	Model 2	RMS	Sec. str.	Mutation
WILD	Seq1	1.115	Loop	Insertion
	Seq2	0.690	Loop	Insertion
	Seq3	1.115	Loop	Insertion
	Seq4	0.533	Loop	Insertion
	Seq5	0.534	Loop	Insertion
	Seq6	1.107	Loop	Insertion

Seq2	Seq1	0.246	Loop	
	Seq3	0.246	Loop	
	Seq4	0.247	Loop	
	Seq5	0.256	Loop	
	Seq6	0.135	Loop	

Seq5	Seq4	0.013	Loop	

## Data Availability

The data used to support the findings of this study are included within the article.
